# Misclassification of causes of death among a small all-autopsied group of former nuclear workers: Death certificates vs. autopsy reports

**DOI:** 10.1371/journal.pone.0302069

**Published:** 2024-05-03

**Authors:** Stacey L. McComish, Xirui Liu, Florencio T. Martinez, Joey Y. Zhou, Sergey Y. Tolmachev

**Affiliations:** 1 United States Transuranium and Uranium Registries, College of Pharmacy and Pharmaceutical Sciences, Washington State University, Richland, Washington, United States of America; 2 Office of Domestic and International Health Studies, United States Department of Energy, Washington, District of Columbia, United States of America; Zahedan University of Medical Sciences, ISLAMIC REPUBLIC OF IRAN

## Abstract

The U.S. Transuranium and Uranium Registries performs autopsies on each of its deceased Registrants as a part of its mission to follow up occupationally-exposed individuals. This provides a unique opportunity to explore death certificate misclassification errors, and the factors that influence them, among this small population of former nuclear workers. Underlying causes of death from death certificates and autopsy reports were coded using the 10^th^ revision of the International Classification of Diseases (ICD-10). These codes were then used to quantify misclassification rates among 268 individuals for whom both full autopsy reports and death certificates with legible underlying causes of death were available. When underlying causes of death were compared between death certificates and autopsy reports, death certificates correctly identified the underlying cause of death’s ICD-10 disease chapter in 74.6% of cases. The remaining 25.4% of misclassified cases resulted in over-classification rates that ranged from 1.2% for external causes of mortality to 12.2% for circulatory disease, and under-classification rates that ranged from 7.7% for external causes of mortality to 47.4% for respiratory disease. Neoplasms had generally lower misclassification rates with 4.3% over-classification and 13.3% under-classification. A logistic regression revealed that the odds of a match were 2.8 times higher when clinical history was mentioned on the autopsy report than when it was not. Similarly, the odds of a match were 3.4 times higher when death certificates were completed using autopsy findings than when autopsy findings were not used. This analysis excluded cases where it could not be determined if autopsy findings were used to complete death certificates. The findings of this study are useful to investigate the impact of death certificate misclassification errors on radiation risk estimates and, therefore, improve the reliability of epidemiological studies.

## Introduction

The United States Transuranium and Uranium Registries (USTUR) is a research program that is designed to study the biokinetics of actinides, such as plutonium and americium, in the human body, and to apply data in support of radiation epidemiological studies. Registrants are volunteers who had a history of work at facilities that handled actinide elements, and who donated their organs and tissues, or entire bodies, to the USTUR for postmortem research. The USTUR routinely performs an autopsy on each Registrant, and obtains death certificates. All-autopsied populations such as this are advantageous, because they avoid biases associated with the scenarios that often lead to autopsies, such as suspicious or unexpected deaths and ambiguous causes of death. This provides a unique opportunity to study the accuracy of cause of death information in this small, all-autopsied group of workers from nuclear facilities.

It is well established that misclassification errors occur on death certificates, when compared to autopsy reports [1–8]. Match rates with autopsy reports have been reported to range from approximately 50% to 80%, where higher levels of agreement were associated with matches at the level of broad disease categories such as neoplasms. Gold and Kathren [9] previously explored the accuracy of death certificate causes of death among the USTUR’s first 260 deceased donors. They observed that of the 127 Registrants for whom the USTUR had both an autopsy report and a death certificate, 89% had a good or complete match between the causes of death on the autopsy report and the death certificate. Gold and Kathren observed that their rate of agreement was higher than would have been expected based on studies of the general population. They suggested that the high rate of agreement between autopsy findings and death certificates may have occurred because attending physicians frequently knew that Registrants were involved in a research program. Therefore, physicians may have considered causes of death more carefully or been more likely to use autopsy findings to assign the cause of death. Since the Gold and Kathren paper was published, additional Registrants have passed away and an effort was made to obtain as many missing death certificates as possible. These data can be used to look more closely at the accuracy of cause of death statements on Registrant death certificates by comparing them to autopsy reports as a “gold standard,” and to explore factors that may influence the rate of agreement. This study also uses data from death certificates to separate them into groups based upon whether the autopsy findings were used to certify causes of death, and explores the impact this has on match rates.

The International Classification of Diseases, Revision 10 (ICD-10) was used in this study to assess the level of agreement between death certificates and autopsy reports. ICD-10 classifies diseases and other health problems using an alphanumeric coding structure [10], which can be used to compare the underlying causes of death (UCODs) on death certificates to UCODs on autopsy reports. It is broken into 22 broad disease chapters. The first digit of each code is a letter, which is followed by two numeric characters. These three-characters form categories that represent either single conditions or groups of diseases with common characteristics.

## Materials and methods

This study was performed as a part of the USTUR research program, which was reviewed and approved by the Central Department of Energy Institutional Review Board No. WASU-68-50181. The USTUR has continuously been in operation since 1968, when Registrant recruitment was initiated. Registrants, or appropriate family members, were required to sign a written authority for autopsy and a release of medical records. In addition to these forms, a formal informed consent form has been required for many years. While Registrants are no longer actively recruited, new Registrants may be accepted into the program if they received at least 74 Bq of internally deposited radionuclides and/or 100 mSv cumulative external dose. Due to the ongoing and long-term nature of the USTUR research program, death certificates and autopsy reports were acquired between approximately 1968 and 2020. Researchers have had continual access to these records throughout the course of this misclassification study, which was initiated in 2019.

### Autopsy report elements

Autopsies were performed on all USTUR Registrants; however, some autopsy reports were missing, and the amount of information provided by pathologists on the 314 available autopsy reports varied from a full, multipage report with a summary of the pathologist’s findings to a one-page list of diagnoses. In order to better understand the completeness of autopsy reports, and their limitations as a “gold standard,” seven autopsy report elements were defined (Table 1), and autopsy reports were inspected to see which elements they contained. The presence of gross internal findings was used to define a full autopsy report for the misclassification analysis. As such, gross internal findings were required to include descriptions of multiple organs or organ systems, and not just an isolated description of the lungs, for example, in an individual that died from lung cancer. However, an alternative format was accepted where the pathologist listed all organs that were inspected, and elaborated only on abnormal findings.

**Table 1:** Autopsy report elements.

Element	Definition	Examples
List of diagnoses	A list of pathological diagnoses, which sometimes incorporated additional clinical findings. Typically found in a bulleted format or a list.	Provisional diagnoses, final pathological diagnoses, autopsy findings.
Clinical	Any information about what happened prior to death, regardless of how brief or detailed.	Clinical history section, mention of surgery in the list of diagnoses, statement that “he fell at home”
Summary	A summary of the autopsy findings, ranging from a brief identification of the cause of death to a page-long narrative about diseases present at death in relation to clinical findings.	Summary, opinion, clinicopathologic summary
Cause of death section	A section labelled “cause of death,” or “cause of death” in parentheses next to an item in the list of diagnoses.	Immediate cause of death, underlying cause of death
Gross external findings	Any description of the exterior of the body, ranging from a few sentences to a detailed description.	External examination, evidence of injury, weight, scars, tattoos, unembalmed, rigor mortis
Gross internal findings^a^	Gross descriptions of internal findings for various organs or organ systems, typically multiple pages long.	Internal examination, gross protocol
Microscopic findings	Any description of histological findings. Did not include a list of sections taken to make slides if there was no comment on the findings.	Microscopic description, microscopic examination

^a^Used to define a full autopsy report for the misclassification analysis.

### Case selection for misclassification analysis

**Fig 1:**
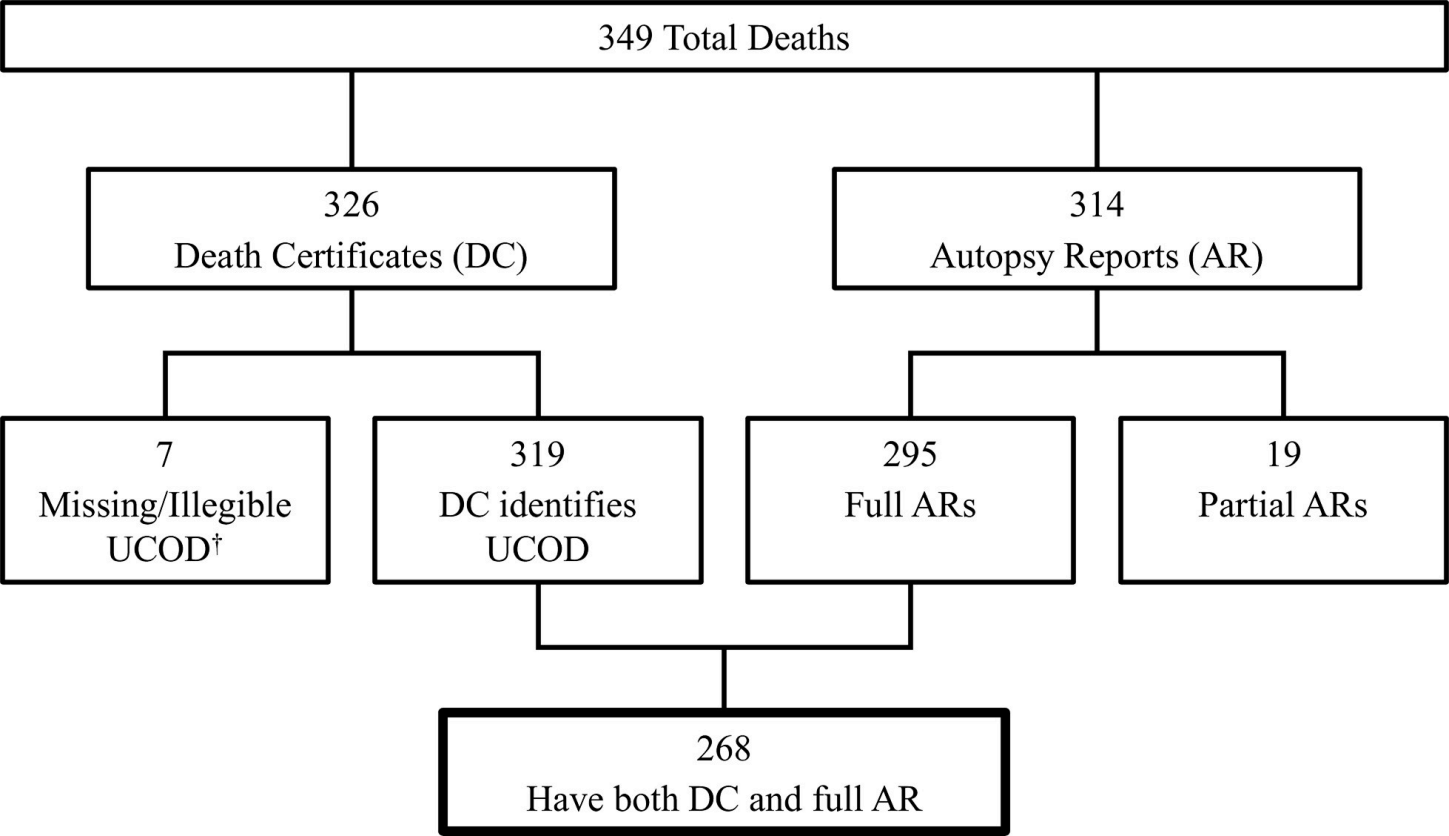
Case selection. ^†^UCOD = Underlying Cause of Death.

Out of 349 Registrants who died on or before December 31, 2020, death certificates that contained legible UCODs were available for 319 individuals. Six additional Registrants had death certificates that were missing the cause of death section, and one death certificate was illegible. Out of the 314 available autopsy reports, 295 were full autopsy reports. The remaining 19 reports did not contain gross descriptions of internal findings for various organs or organ systems, and were excluded from the analysis. Eighty-two percent of the 295 full autopsy reports clearly identified the underlying cause of death, and the remaining 18% provided a list of diagnoses to summarize the findings. When full autopsy reports were compared to the death certificates that contained a legible UCOD, a subset of 268 individuals was identified and used to determine rates of misclassification of diseases on death certificates (Fig 1). Individuals in the study group were predominantly white males who passed away between 1969 and 2020 (Table 2).

**Table 2:** Study group demographics.

Characteristic	Frequency (%)
Sex	
Male	260 (97.0%)
Female	8 (3.0%)
Race	
White	265 (98.9%)
Other	3 (1.1%)
Age, years	
25–60	51 (19.0%)
60–79	149 (55.6%)
80–96	68 (25.4%)
Mean ± SD	71.1±13.8 y
Year of death	
1969–1979	65 (24.3%)
1980–1999	128 (47.8%)
2000–2020	75 (28.0%)
Mean ± SD	1991±14

### Medical coding

Registrant death certificates were sent to a professional nosologist who identified the UCOD and coded it using the ICD-10. The same nosologist had previously coded all conditions on the autopsy reports. It is important to note that the death certificates and autopsy reports were ICD coded on different occasions. Thus, when the nosologist identified the UCOD from the death certificates, she was unaware of the autopsy findings. A medical doctor (MD) reviewed each autopsy report, determined the UCOD, and identified the previously documented ICD-10 code associated with the UCOD. The cause of death sections and summaries from the autopsy reports were the most common sources for UCOD information, though the autopsy reports as a whole, including the lists of diagnoses, were considered. Three autopsy reports were not coded by the nosologist, so the MD assigned the ICD-10 code to the UCOD for these individuals.

### Misclassification analysis

Each case was assigned a match status by comparing the UCOD on the death certificate to the UCOD from the autopsy report. If both UCODs belonged to the same ICD-10 disease chapter, the case was coded as a match. If they belonged to different chapters, it was coded as a mismatch.

Due to the small number of cases, analysis focused on the most common causes of death. According to the death certificates, five ICD-10 chapters had at least 10 cases: circulatory, neoplasms, respiratory, external causes, and nervous system. These chapters were used in the misclassification analysis. The remaining cases formed the “Other” disease category, which consisted of all diseases with less than 10 observations. The misclassification analysis assumed that autopsy reports provided the true UCODs, and could be used to assess the accuracy of death certificates. Thus, a false positive meant that the death certificate stated that an individual died of a disease, when the autopsy report indicated that they did not. Similarly, a false negative meant that the death certificate indicated that an individual did not die of a disease, when the autopsy report indicated that they did.

Match rates were calculated from the number of true positives, i.e. matches, on the death certificates using Eq (1). Over- and under-classification rates for each ICD-10 chapter were calculated from the number of false positives and false negatives on the death certificates using Eq (2) and Eq (3), respectively. The over-classification rate, which is also known as the false positive rate, quantified how frequently a death certificate indicated that an individual died of a certain disease, when they did not die of that disease. The under-classification rate, which is also known as the false negative rate, quantified how frequently a death certificate indicated that a person did not die of a disease in a certain ICD-10 chapter, when they actually died of a disease in that chapter. The “Other” disease category was excluded from the calculations of over- and under-classification due to the pooled nature of the data. When multiple disease chapters were pooled, cases that were a mismatch from one chapter in the “Other” category to a different chapter in the “Other” category would have been considered a match, even if the UCOD on the death certificate was very different from that on the autopsy report.


$$Match\;rate=\frac{True\;Positives}{True\;Positives+False\;Positives}$$ (1)

$$Over‐classification\;rate=\frac{False\;Positives}{False\;Positives+True\;Negatives}$$ (2)

$$Under‐classification\;rate=\frac{False\;Negatives}{False\;Negatives+True\;Positives}$$ (3)

Cases were divided into three groups to investigate the influence of autopsy findings on the match status: (1) autopsy report was used to determine the death certificate UCOD, (2) autopsy report was not used, and (3) unknown. Initially, cases were assigned to an autopsy influence group based upon two items on the death certificate, which asked (1) if an autopsy had been performed, and (2) if the autopsy findings were available or considered in determining the cause of death (Table 3). Corrections to the initial autopsy influence groups were made based on the logic check that if a cause of death was certified on the death certificate before the autopsy had even been performed, it could not have been influenced by autopsy findings. Thus, four cases from the “Used” group and 17 from the “Unknown” group were moved to the “Not Used” group. The match rate for the 21 cases that were moved to the “Not Used” autopsy influence group was approximately the same as the match rate for the entire “Not Used” group, which confirmed the validity of the decision to reclassify the cases. Once the autopsy influence groups had been finalized, over- and under-classification rates were calculated for each group using Eq (2) and Eq (3).

**Table 3:** Autopsy influence groups.

Group	Autopsy performed	Autopsy findings considered
Used	Yes	Yes
Not Used	No	Blank/Missing/No/n.a.
	Yes	No
Unknown	Yes	Blank/Missing
	Blank/Missing	Blank/Missing

A logistic regression was performed using RStudio [11] to evaluate the influence of different factors on the match rate. Independent variables for this analysis were: the “Not Used” and “Used" autopsy influence groups; clinical history and no clinical history; and the five most common ICD-10 disease chapters, plus the “Other” disease category. The neoplasms group was used as the reference disease category, and Registrants in the “Unknown” autopsy influence group were excluded from this analysis to avoid introducing noise into the comparison of the “Not Used” and “Used” autopsy influence groups.

## Results

### Autopsy report elements

Table 4 provides the frequency of each autopsy report element for full reports, incomplete reports, and all reports combined. Overall, the most common autopsy report elements were: list of diagnoses (96.8%), gross external findings (93.9%), gross internal findings (93.9%) and microscopic findings (92.0%). The least common element was a cause of death section (25.2%), whose frequency was much lower than the frequency of other elements.

**Table 4:** Frequency of autopsy report elements.

Element	Full Reports (*n* = 295)	Incomplete (*n* = 19)	Total (*n* = 314)
List of diagnoses	96.9%	94.7%	96.8%
Clinical	71.2%	52.6%	70.1%
Summary	70.2%	42.1%	68.5%
Cause of death section	24.7%	31.6%	25.2%
Gross external findings	99.7%	5.3%	93.9%
Gross internal findings	100.0%	0.0%	93.9%
Microscopic findings	95.9%	31.6%	92.0%

### Misclassification analysis

When death certificate and autopsy report UCODs were compared, 74.6% matched. Table 5 provides details about the number of matches and mismatches among the full 268-individual dataset. Matches are indicated in bold along the diagonal. Mismatches are on the off-diagonal, and indicate how UCODs from death certificates differed from those reported by autopsy reports. ICD-10 chapters that represented diseases from which USTUR Registrants did not die were excluded.

**Table 5:** Number of cross-classified UCODs from death certificates and autopsy reports^a^.

	ICD-10 Chapter^b^		Autopsy Report UCOD															
			1	2	3	4	5	6	9	10	11	13	14	17	18	19	20	Total
Death Certificate UCOD	1	Infectious and parasitic	–	–	–	–	–	–	2	–	–	–	–	–	–	–	–	2
	2	Neoplasms	–	**72**	1	–	–	–	5	2	–	–	–	–	–	–	–	80
	3	Blood and immune	–	–	**1**	–	–	–	–	–	1	–	–	–	–	–	–	2
	4	Endocrine, nutritional, metabolic	–	–	–	**1**	–	–	1	–	–	–	–	–	–	–	–	2
	5	Mental and behavioral	–	1	–	–	**1**	1	3	1	–	–	–	–	–	–	–	7
	6	Nervous system	–	–	–	–	–	**10**	4	1	–	–	–	–	–	–	–	15
	9	Circulatory	1	3	–	1	–	2	**90**	5	1	–	1	1	1	1	1	108
	10	Respiratory	–	4	–	–	–	–	12	**10**	–	–	–	–	–	–	–	26
	11	Digestive	–	–	–	–	–	–	–	–	**2**	–	–	–	–	–	–	2
	13	Connective tissue	–	–	–	–	–	–	1	–	–	–	1	–	–	–	–	2
	14	Genitourinary	–	1	–	–	–	–	2	–	–	–	**1**	–	–	–	–	4
	17	Malformations	–	–	–	–	–	–	–	–	–	–	–	–	–	–	–	0
	18	Symptoms and findings	–	1	–	–	–	–	1	–	1	–	–	–	–	–	–	3
	19	Injury or poisoning	–	–	–	–	–	–	–	–	–	–	–	–	–	–	–	0
	20	External causes	–	1	–	–	–	–	–	–	–	–	–	–	–	2	**12**	15
		Total	1	83	2	2	1	13	121	19	5	0	3	1	1	3	13	268

^a^Matches are shown on the diagonal in bold

^b^Several ICD-10 chapters were excluded, because no Registrants died from diseases in those chapters.

### Most common diseases

Five ICD-10 chapters had 10 or more UCOD observations on the death certificates: circulatory (40.3%), neoplasms (29.9%), respiratory (9.7%), external causes (5.6%), and nervous system (5.6%). The remaining Registrants died from other causes (9.0%). Metrics for diseases in these chapters are given in Table 6. The match rate between UCODs on death certificates and UCODs on autopsy reports at the ICD-10 chapter level ranged from 38.5% for respiratory diseases to 90.0% for neoplasms. The over-classification rate ranged from 1.2% for external causes to 12.2% for circulatory disease, and the under-classification rate ranged from 7.7% for external causes of death to 47.4% for respiratory disease.

**Table 6:** Chapter matches, and over- and under-classification rates^a^ for the five most common disease categories.

ICD-10 Chapters		*n* ^b^	Matches	Over-	Under-
2	Neoplasms	80	72 (90.0%)	8 (4.3%)	11 (13.3%)
6	Nervous system	15	10 (66.7%)	5 (2.0%)	3 (23.1%)
9	Circulatory	108	90 (83.3%)	18 (12.2%)	31 (25.6%)
10	Respiratory	26	10 (38.5%)	16 (6.4%)	9 (47.4%)
20	External causes	15	12 (80.0%)	3 (1.2%)	1 (7.7%)
	Other	24	6 (25.0%)	n/a	n/a
	All Causes	268	200 (74.6%)	n/a	n/a

^a^Number of false positives and false negatives, with the corresponding over- and under-classification rates in parentheses.

^b^Number of death certificates with UCODs in each chapter.

When the dataset was divided into three autopsy influence groups, 114 (42.5%) death certificates did not use autopsy findings, 53 (19.8%) used autopsy findings, and the influence of the autopsy findings was unknown for 101 (37.7%) cases. Table 7 summarizes the match rates for each autopsy influence group. Overall, the match rate was higher for the “Used” group than the “Not Used” group. This trend held true for all five of the most common disease chapters. However, no clear pattern emerged for the “Unknown” autopsy influence group: the “Unknown” group was lower than the “Not Used” group for neoplasms, but higher than the “Used” group for circulatory disease.

**Table 7:** Chapter matches by autopsy influence groups.

		Not Used		Used		Unknown	
ICD-10 Chapter		*n* ^a^	Matches	*n* ^a^	Matches	*n* ^a^	Matches
2	Neoplasms	27	25 (92.6%)	14	14 (100%)	39	33 (84.6%)
6	Nervous System	11	6 (54.5%)	1	1 (100%)	3	3 (100%)
9	Circulatory	44	33 (75.0%)	24	20 (83.3%)	40	37 (92.5%)
10	Respiratory	13	5 (38.5%)	3	2 (66.7%)	10	3 (30.0%)
20	External Causes	5	3 (60.0%)	5	5 (100%)	5	4 (80.0%)
	Other	14	1 (7.1%)	6	4 (66.7%)	4	1 (25.0%)
	All Causes	114	73 (64.0%)	53	46 (86.8%)	101	81 (80.2%)

^a^Number of death certificates with UCODs in each chapter.

When over- and under-classification rates were calculated (Table 8), the “Used” autopsy influence group almost always had lower misclassification rates than the “Not Used” group. For example, both over- and under-classification of neoplasms was higher in the “Not Used” group than in the “Used” group. The”Not Used” autopsy influence group is more representative of death certificates in the general population, where autopsies are performed only a small fraction of the time. For death certificates that did not use autopsy findings, over-classification rates ranged from 1.8% for external causes to 18.0% for circulatory disease. Under-classification rates among certificates that did not use autopsy findings ranged from 21.9% for neoplasms to 50.0% for respiratory disease.

**Table 8:** Over- and under-classification rates^a^ by autopsy influence groups.

ICD-10 Chapter		Not Used		Used			Unknown		
		Over-	Under-		Over-	Under-		Over-	Under-
2	Neoplasms	2 (2.4%)	7 (21.9%)		0 (0.0%)	2 (12.5%)		6 (9.1%)	2 (5.7%)
6	Nervous System	5 (4.8%)	3 (33.3%)		0 (0.0%)	0 (0.0%)		0 (0.0%)	0 (0.0%)
9	Circulatory	11 (18.0%)	20 (37.7%)		4 (12.1%)	0 (0.0%)		3 (5.7%)	11 (22.9%)
10	Respiratory	8 (7.7%)	5 (50.0%)		1 (2.0%)	2 (50.0%)		7 (7.3%)	2 (40.0%)
20	External Causes	2 (1.8%)	1 (25.0%)		0 (0.0%)	0 (0.0%)		1 (1.0%)	0 (0.0%)

^a^Number of false positives and false negatives, with the corresponding over- and under-classification rates in parentheses.

The logistic regression results (Table 9) indicated that when death certificate certifiers used autopsy findings to determine the causes of death, the odds that the death certificate and autopsy report UCODs would match increased significantly. Similarly, when clinical history was mentioned in autopsy reports, the odds of a match increased significantly. Additionally, compared to death certificates where the individuals’ UCODs were neoplasms, the odds of a match decreased significantly when the reported UCOD was a circulatory disease, a respiratory disease, a disease of the nervous system or in the “Other” category of diseases.

**Table 9:** Logistic regression results–influence of autopsy use, clinical history and disease chapters on the match rate.

Coefficient	Estimate	Odds Ratio	*p*-value	sig
Autopsy report "Used"	1.22	3.4	0.0257	*
Clinical history in autopsy report	1.02	2.8	0.0191	*
ICD-10 disease chapters				
Neoplasms	Reference category			
Circulatory	-1.69	0.18	0.0339	*
Respiratory	-3.08	0.046	0.0008	***
External causes	-1.81	0.16	0.101	
Nervous system	-2.58	0.076	0.00727	**
Other chapters	-4.33	0.013	<0.0001	***

* *p* ≤ 0.05

** *p* ≤ 0.01

*** *p* ≤ 0.001

## Discussion

The overall match rate found in this study (74.6%) was lower than Gold and Kathren’s initial findings for USTUR Registrants (89%). The match rate for the entire dataset is likely lower than Gold and Kathren’s match rate due to different post-mortem protocols at the Registries. In approximately 1992, testing for HIV and hepatitis became a routine procedure at the USTUR [12]. It required a negative test result before the autopsy could be conducted. This frequently delayed the autopsy by two or more days, such that some physicians may have been required to certify death certificates before the autopsy findings became available. Thus, the match rate likely decreased in recent years, because fewer death certificates were informed by autopsy findings. Indeed, Registrant data supports this observation. When cases in the unknown autopsy influence group were excluded, 49% of death certificates filed prior to 1992 used autopsy findings, but only 20% filed during or after 1992 used autopsy findings.

The "Not Used" autopsy influence group should be most representative of the general population, where information from autopsies typically is not used to revise death certificates [4]. The match rate for all causes of death in the “Not Used” autopsy influence group (64.0%) was on the low end of previously published values [1–8], given that one of the previously published match rates (53%) [7] was notably lower than the range found by other studies (68%-81%). The higher match rate found by other studies may indicate that the studies included some death certificates that used autopsy findings, or that they employed different methodologies for using autopsy findings to assess the accuracy of death certificates.

The existence of death certificate misclassification errors is well documented [1–8, 13]. A review article by Roulson et al. [13] discussed two papers that assessed trends in death certificate errors over time. While some diseases showed an improvement in diagnostic sensitivity over time, others showed no improvement or a decline. Overall, little to no improvement in the rate of discrepancies was observed. Death certificate errors can arise from undiagnosed diseases discovered at autopsy [13], as well as from incorrect reporting of causes of death on death certificates [14–16]. For example, McGivern et al. compared the ICD coding for original Vermont death certificates to the coding for mock certificates that had been prepared based on clinical summaries from medical records. Out of 580 original death certificates, 348 (60%) contained a difference between the original death certificate and the mock certificate that would have changed the UCOD. Common death certificate errors involving causes of death include: listing general conditions instead of specific conditions, a sequence of events leading to death that was out of order or did not make sense, and reporting the wrong cause of death [17, 18]. Statutory authority for certifying death certificate causes of death have varied by state and through time, and may contribute to inaccuracies in cause of death statements since the accuracy of UCODs vary among different types of certifiers, such as attending physicians or medical examiners [19].

A logistic regression was used to explore the significance of factors, such as autopsy influence, that were associated with lower match rates between death certificates and autopsy reports. Initially, the logistic regression model included age at death and year of death, along with the “Used” autopsy influence group and clinical history as independent, binary variables. However, age at death, year of death, and autopsy influence were highly correlated with each other due to the previously mentioned HIV testing, and the fact that a disproportionate number of Registrants were recruited during the 1970s and 1980s. Since 91% of Registrants were enrolled prior to 1992, those who passed away after 1992 tended to be older (81.1 ± 9.6 y) than those who passed away before 1992 (average: 63.0 ± 11.1 y). Therefore, age is highly correlated with year of death, and year of death is correlated with autopsy influence group. To avoid multicollinearity in the logistic regression model, age at death and year of death were removed from the regression. The logistic regression indicated that when autopsy reports were used to determine death certificate causes of death, the odds that the UCOD on the two documents would match significantly increased, with an odds ratio of 3.4. This association was also observed for over- and under-misclassification rates, where misclassification rates were almost always lower in the “Used” group than in the “Not Used” group. This result is not surprising; however, it highlights the importance of taking autopsy influence into consideration when using misclassification rates to evaluate or correct underlying cause of death ascertainment errors. To our knowledge, few other studies have investigated the potential impact of the use of autopsy findings on death certificate accuracy. Engel et al.’s [2] analysis of records from 257 autopsied individuals did not investigate the use of autopsy findings, but they did compare death certificates that indicated that an autopsy had been performed to those that did not. The authors found no association between misclassification on death certificates and indication that an autopsy had been performed. Schottenfeld et al. [8] used autopsy findings from two community hospitals to determine how often death certificates contained inaccuracies that required recoding of the UCOD. One hospital routinely corrected death certificates using autopsy findings, and the other did not. Death certificates from the hospital that did not correct its death certificates needed to be recoded more frequently (24%) than those from the hospital that corrected its death certificates (10%).

The logistic regression also found that the odds of a match were significantly lower for circulatory, respiratory, nervous system, and other causes of death when compared to neoplasms as a reference category. This was not surprising, given that advanced neoplastic disease is often easily identifiable at autopsy, and the disease may follow a clear clinical progression from diagnosis to death. Additionally, neoplasms are less likely to be associated with multiple causes of death on the death certificates than are chronic diseases. D’Amico et al. [20] studied 372 death certificates from Naples, Italy and found that neoplasms were listed in association with other causes less frequently (16.8% of cases) than were chronic bronchitis (74.1%) or hypertension (69.2%). When chronic diseases, such as those of the respiratory and circulatory system, occur simultaneously with other causes, selection of a single cause for the UCOD becomes less straightforward.

In this study, autopsy influence could not be determined for 38 percent of death certificates. This is largely a consequence of changes to death certificates during the three decades represented by this study. The current U.S. Standard Death Certificate [21] includes two items that shed light on the extent to which autopsy findings may have influenced certified causes of death. The first, which has been continually included on the standard certificate since 1949, asks if an autopsy was performed. The second item, which has not been consistently included on the standard certificate, asks: “were autopsy findings available to complete the cause of death?” Inconsistencies among revisions of the U.S. Standard Death Certificate appear to have translated to inconsistent inclusion of these items on state certificates. While 97% of death certificates used in this study, representing 28 states, asked if an autopsy had been performed, only 59% asked if the autopsy findings had been used to determine the cause of death. The latter item was introduced on the standard certificate in 1968 with different wording, removed in 1978, and reintroduced in 1989 with wording that was similar to the current wording [22]. Death certificates from USTUR Registrants indicated that individual states’ responses to these changes varied. Colorado followed a similar pattern to the U.S. standard certificate, New Mexico did not remove the item in 1978, and Washington removed it shortly after 1978, but did not reinstate it until approximately 2004. These three states are the three most common states of death for individuals in this study.

One limitation of this study is the accuracy of autopsy reports themselves. In this study, autopsy reports have been treated as a “gold standard” that can be used to definitively identify the true underlying cause of death. While autopsy reports generally provided a more detailed picture of the diseases present at the time of death, they were still an imperfect source of information. There are several reasons for this. Only 24.7% of autopsy reports contained a cause of death section, and rarely did the autopsy reports lay out the progression from the underlying cause of death to the immediate cause of death using language that directly translated to the structure on the death certificates. While the process of inferring the underlying cause of death was often straightforward–for example, in the case of a metastatic neoplasm–it could also require subjective judgement when multiple conditions identified on the autopsy reports had to be weighed against each other to select a single UCOD. The impact of this ambiguity can be seen in the “Used” autopsy influence group, where our medical doctor and the certifying physician identified different UCODs for seven cases in the “Used” autopsy influence group (Table 7), despite the certifying physician’s indication that autopsy findings were considered when completing the death certificate.

Another factor that may limit the reliability of autopsy reports as a “gold standard” is a pathologist’s lack of access to clinical history. For 30% of the 268 Registrants in this study, the autopsy reports made no mention of events or conditions prior to death, and it may be that a significant portion of these autopsy reports were written without the benefit of clinical information. Without clinical history, it may be difficult for the pathologist to rank the importance of observed anatomic abnormalities in relation to death, particularly when multiple disease processes were present. Indeed, the logistic regression indicated that clinical history had a statistically significant influence on match rate in the USTUR population, and the odds ratio for clinical history mentioned on the autopsy report was 2.8. This highlights the importance of providing pathologists with relevant clinical information prior to an autopsy and summarizing clinical history in autopsy reports [23, 24].

Additional limitations of this study include the small population size, and unique factors associated with USTUR operations. The potential impact of HIV testing on match rates has already been discussed. Another factor unique to the USTUR is the amount of time between consent and death. A noteworthy minority (14%) of individuals became Registrants on or after the day they passed away. In contrast to those who passed away many years after agreeing to have an autopsy, these individuals, or the next of kin who signed their consent paperwork, likely knew the cause of death and/or circumstances surrounding death when they became Registrants. According to a logistic regression, becoming a Registrant on or after the day of death was associated with a significant increase in match rate (*p* = 0.020). It is difficult to say if this is a consequence of knowing what the cause of death was, or if it was due to other factors such as better communication among certifying physicians, pathologists, and/or Registries staff. The most notable characteristics of individuals who registered on or after the day of death were that most of them worked at Rocky Flats (83%) and most died prior to 1990 (average year of death: 1980). Individuals who became Registrants on or after their day of death were also more likely to be in the “Used” autopsy influence group, which is associated with a higher match rate and may suggest better communication played a role. Disease category does not appear to have increased the match rate.

An interesting question is that of why 18% of autopsy reports did not clearly indicate the underlying cause of death. Clearly identified UCODs were typically found in a summary of findings, such as a clinicopathological summary, or a cause of death statement. Summaries were only included on 69% of USTUR autopsy reports, and only 25% of reports had a statement clearly labelled “cause of death,” which sometimes indicated only the immediate cause of death. Possible explanations include a lack of information about the individuals’ clinical histories, multiple contributing causes of death typically associated with advanced age [20, 25], and pathologists’ personal reporting styles. A closer look at the autopsy reports did not support the idea that a lack of clinical history prevented pathologists from clearly identifying the UCOD. Neither does it appear that it was a consequence of advanced age, given that the age of Registrants whose autopsy reports did not specify the UCOD (65.7 ± 11.4) was no greater than that of Registrants whose reports did specify the UCOD (72.2 ± 14.0). However, it does appear that certain pathologists tended to clearly identify the UCOD in their autopsy reports, while others provided only a list of diagnoses and pathological observations. For example, five pathologists wrote 71% of the 93 autopsy reports from the state of Washington. Four of these pathologists always stated the UCOD in their reports, and the fifth pathologist only omitted it on one occasion. The next most frequently used pathologist, who wrote 4% of Washington autopsy reports, provided only a list of diagnoses and/or autopsy findings on three of his four autopsy reports.

Effort is often invested into improving the reliability of epidemiological findings by improving dose estimates. However, the quality of mortality data is also important. Former nuclear workers from Department of Energy facilities represent a major target population for US radiation epidemiological studies [26, 27]. Since USTUR Registrants are former nuclear workers who worked similar jobs during similar time periods to those included in epidemiological studies, the under- and over- classification rates identified in this study can be used to investigate the impact of death certificate misclassification errors on radiation risk estimates.

While assessing the impact of the misclassification errors is beyond the scope of this work, preliminary calculations indicate that match rate does not appear to be dose dependent. Full dose assessments for internally incorporated actinides are time consuming and have not been completed for all USTUR Registrants; however, there are several indicators of dose that can be used to check for possible associations between match rate and dose: external dose, terminal dose rate to the liver (TDR_Liver_), and terminal dose rate to the lungs TDR_Lungs_. The terminal dose rate is the dose rate to an organ at the time of death (mGy/y), and is calculated directly from the concentration of actinides radiochemically measured in that organ postmortem. TDRs are imperfect indicators of relative internal doses, due to differences in the absorption of soluble versus insoluble actinides from the lungs. However, TDR_Liver_ is a useful indicator of systemic uptake of actinides, and TDR_Lungs_ provides information about the amount of an actinide that was retained in the lungs long term. The possibility that match rate could be dose dependent was explored using three linear regressions: match status vs. external dose, match status vs. TDR_Liver_, and match status vs. TDR_Lung_. As expected, no significant relationship between match rate and any of the three indicators of dose was observed; thus, it appears that the relationship between match rate and dose is non-differential.

## Conclusions

This study looked at the prevalence of misclassification errors in an all-autopsied group of individuals who worked at nuclear facilities, the majority of whom had a known history of exposure to actinides. The match rate in this occupational population was 74.6% for all deaths. Misclassified cases resulted in over-classification rates that ranged from 1.2% for external causes to 12.2% for circulatory disease, and the under-classification rates ranged from 7.7% for external causes to 47.4% for respiratory disease. Neoplasms generally had lower rates of misclassification, with 4.3% over-classification and 13.3% under-classification.

Focus was placed on analyzing the influence of autopsy findings on the match rate. Among USTUR Registrants, the match rate was 64.0% when autopsy findings were not used to certify death certificate causes of death. This increased to 86.8% for death certificates that did use autopsy findings. When cases in the unknown autopsy influence group were excluded, a logistic regression showed that the odds of a match were 3.4 times higher when autopsy findings were used to certify death certificate causes of death than when they were not. Similarly, the odds of a match were 2.8 times higher for cases where clinical history was mentioned on the autopsy report than for cases where it was not. Both of these findings emphasize the importance of communicating information between clinical staff, including the certifying physician, and the pathologist.

This study is one of only a few studies comparing causes of death between death certificates and autopsy reports using an all-autopsied population. This is also one of the first studies to evaluate cause of death misclassification on death certificates by autopsy influence categories, and in particular, to define autopsy influence groups using two fields on death certificates that were related to autopsy use. The findings of this study can be used to investigate the impact of death certificate misclassification errors on radiation risk estimates and, therefore, improve the reliability of epidemiological studies.

## Supporting information

S1 Appendix Logistic regression results. (XLSX)
